# Dr. Edward P. Doherty

**Published:** 1908-02

**Authors:** 


Dr. Edward P. Doherty, of Dorchester, N. B., died October 3, 1907, aged 46 years. He graduated from the medical department of the University of Buffalo in 1884, and for six years served as surgeon of the Maritime Penitentiary at Dorchester.

